# Experience with transesophageal echocardiography for mitral valve plasty in the remote stage after esophagectomy with gastric tube reconstruction via the posterior mediastinal route

**DOI:** 10.1186/s40981-023-00625-9

**Published:** 2023-07-21

**Authors:** Yuri Sato, Takaharu Tokita, Junichi Saito, Kazuyoshi Hirota

**Affiliations:** 1 Department of Anesthesiology, Aomori Prefectural Central Hospital, 2-1-1 Higashitsukurimichi, Aomori, 030-8553 Japan; 2 Department of Anesthesiology, Hirosaki University Graduate School of Medicine, 5 Zaifu-cho, Hirosaki, 036-8562 Japan

**Keywords:** Mitral valve plasty, Transesophageal echocardiography, Esophageal cancer, Esophagectomy, Gastric tube reconstruction, Retrosternal route, Complications

## Abstract

A 69-year-old male patient with mitral valve prolapse was scheduled for mitral valve plasty. Sixteen years earlier, he had undergone right open thoracotomy for esophageal cancer with subtotal esophagectomy, cervicothoraco-abdominal three-region dissection, posterior mediastinal tube reconstruction, and cervical anastomosis. Postoperatively, the patient had a treatment- and recurrence-free course, and an upper gastrointestinal endoscopy performed 2 years prior revealed no abnormality. We scheduled a transesophageal echocardiography for mitral valve surgery. We attempted to insert the probe but felt resistance at the height of the mid-thoracic region, and the image quality was poor, so we abandoned the intraoperative diagnosis. The surgery was performed as planned, and when the probe was manipulated again at the time of cardiopulmonary withdrawal, the mitral valve could be observed. The mitral valve was judged to be sufficiently repaired, and the surgery was terminated. There were no complications associated with transesophageal echocardiography.

## Background

Transesophageal echocardiography (TEE) is an essential tool for decision-making in patients undergoing cardiac surgery, especially mitral valve surgery.

The average age of patients undergoing cardiac surgery is increasing, and it is not uncommon for patients with cancer or after cancer surgery to undergo cardiac surgery. However, intraoperative cardiac evaluation via TEE after upper gastrointestinal surgery risks further injury to the gastrointestinal tract [1]. In addition, the quality of TEE images obtained in the upper gastrointestinal tract after surgery can vary unpredictably. We herein report a case in which TEE was used safely for mitral valve plasty in the remote postoperative period of esophagectomy/gastric tube reconstruction and was useful for intraoperative diagnosis and decision-making.

## Case presentation

We obtained written informed consent from the patient for the publication of this case report.

A 69-year-old male patient, height 172 cm and weight 49.7 kg, complained of fatigue on exertion and was diagnosed with mitral valve prolapse. Sixteen years earlier, he had undergone right thoracotomy, cervicothoracic-abdominal three-region dissection, posterior mediastinal canal reconstruction, and cervical anastomosis for esophageal cancer. An upper gastrointestinal endoscopy performed 2 years prior to presentation showed no abnormal findings. Preoperative examination revealed no evidence of recurrence of esophageal cancer or metastasis.

Preoperative transthoracic echocardiography (TTE) showed good mobility of the mitral valve leaflets, but the deviation of the valve leaflets near the anterior commissure caused them to droop toward the left atrium and caused a regurgitation jet toward the left posterior atrial wall during systole. A gastrointestinal surgeon advised that insertion of a TEE probe would not be contraindicated if there had been no problems with the upper gastrointestinal endoscopy, but that the gastrointestinal tract, unlike the esophagus, is not completely airtight, such that more distal images could not be safely obtained in a surgical context. We explained the benefits and risks of intraoperative TEE to the patient and obtained his consent to perform TEE. Since mitral valve plasty requires close observation and evaluation by echocardiography during surgery, intraoperative TEE is performed whenever possible. However, to avoid complications, we minimized probe manipulation and prepared for the use of epicardiac echocardiography should adequate TEE images not be obtainable.

**Fig. 1:**
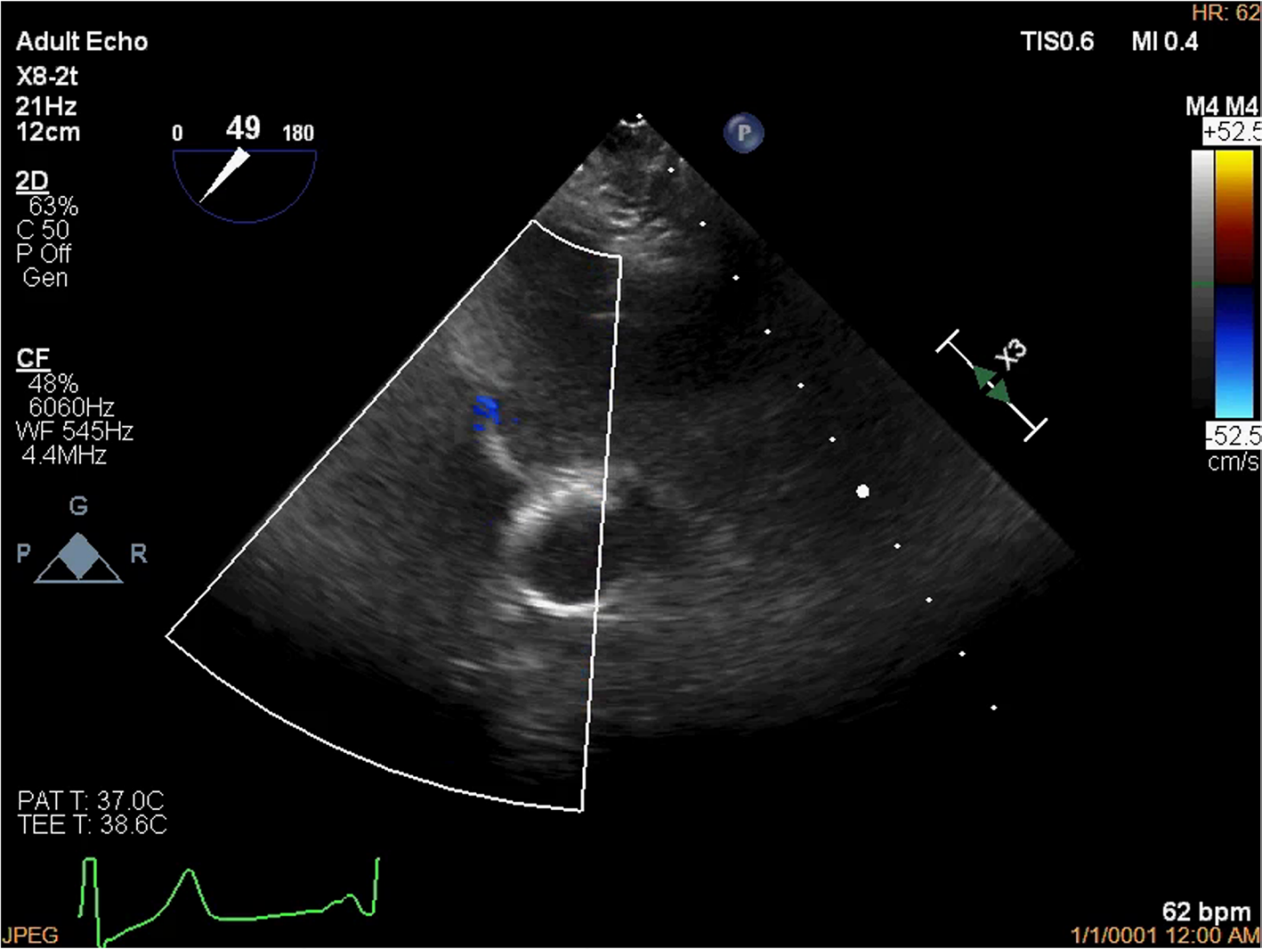
Transesophageal echocardiography image after induction of anesthesia. This is the only transesophageal echocardiography image we recorded after induction of anesthesia. Because of poor image quality due to loose contact of the TEE probe to the reconstructed esophagus mucosa, the aortic valve was barely visible

**Fig. 2:**
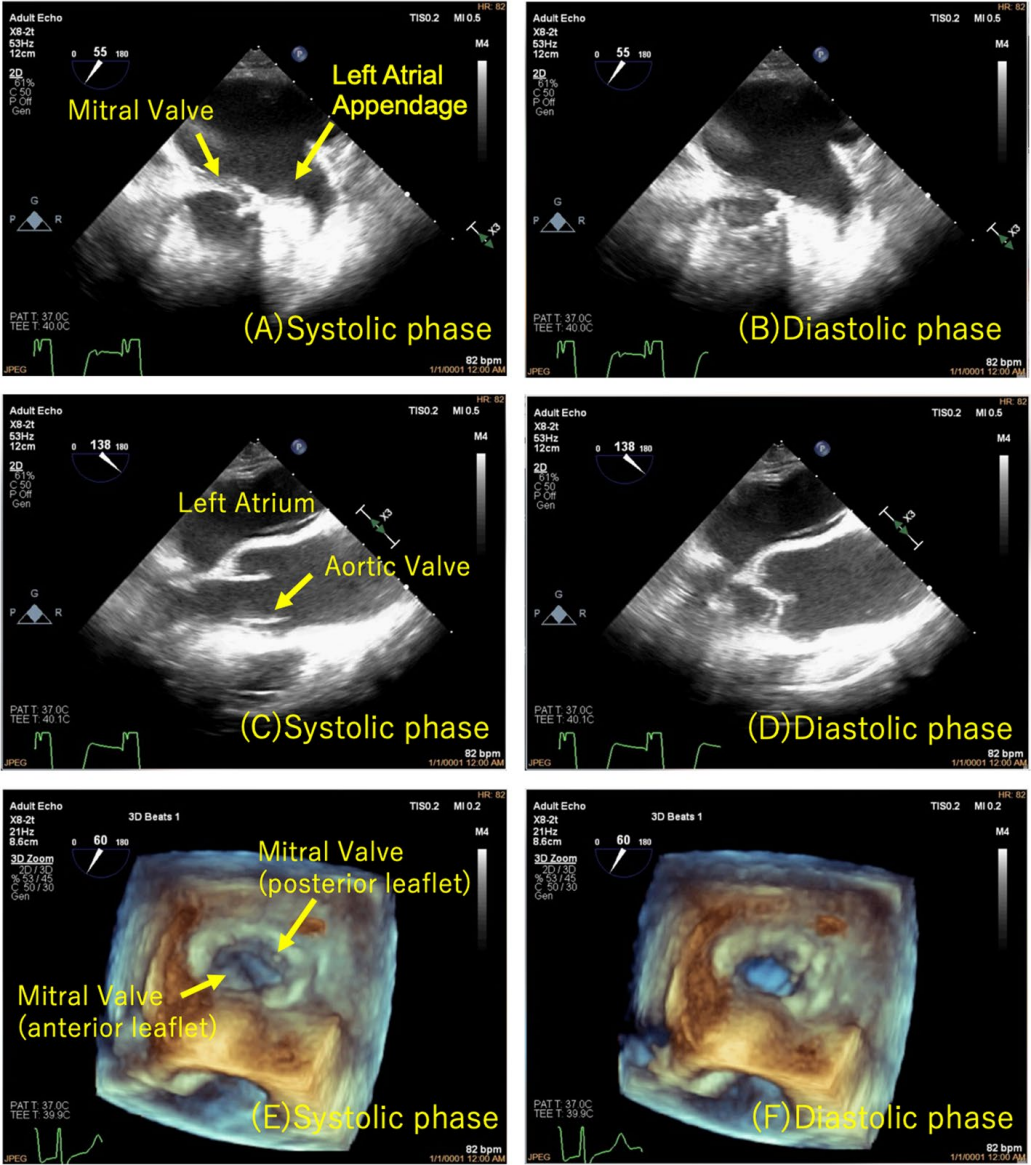
Transesophageal echocardiography during withdrawal from cardiopulmonary bypass (**A** mid-esophageal two-chamber image (systolic phase), **B** mid-esophageal two-chamber image (diastolic phase), **C** mid-esophageal long-axis image (systolic phase), **D** mid-esophageal long-axis image (diastolic phase), **E** 3-dimensional TEE (systolic phase), **F** 3-dimensional TEE (diastolic phase)). During withdrawal from cardiopulmonary bypass, image quality and field of view were improved, and the mitral valve could be observed. No thrombus or large air volume was observed in the left atrium (**A**–**B**), and the mitral valve was deemed sufficiently repaired (**A**–**F**)

After induction of anesthesia, a gentle probe insertion (EPIQ X8-2t shaft diameter 13–14 mm Philips) into the reconstructed esophagus/gastric tube was attempted. Insertion was possible without resistance, but further advancement was abandoned due to resistance at the mid-thoracic level. Because of poor image quality due to loose contact of the TEE probe to the reconstructed esophagus mucosa, the aortic valve was barely visible, and intraoperative evaluation of the mitral valve was deemed impossible (Fig. 1). The TEE probe was placed slightly above the middle esophagus where resistance was felt, and a postoperative cardiac assessment by epicardiac echocardiography was planned. An anterior commissure deviation due to a tendon cord tear was found. After wedge resection suture, mitral annuloplasty with an artificial ring, and tricuspid annuloplasty, the patient was weaned from cardiopulmonary bypass (CPB). The TEE probe was then manipulated again without advancement, image quality and field of view were improved, and the mitral valve could be observed (Fig. 2A–C). Mitral regurgitation had improved to trivial-mild, and no thrombus or large air volume was observed in the left atrium. The mitral valve was deemed sufficiently repaired, and the patient was weaned from CPB. Postoperatively, the patient was extubated in the operating room and admitted to the intensive care unit. There were no symptoms to suggest gastric tube damage, and the patient could drink water without problems. He was transferred to the general ward the day after surgery. TTE performed 6 days after surgery showed trivial mitral regurgitation and no evidence of cardiac dysfunction. Because of the good postoperative course, the patient was discharged on postoperative day 10.

## Discussion

This case indicated that TEE could be used safely in the remote postoperative period of esophageal cancer, and that, although the extent of observation with TEE was limited, it was nonetheless useful for intraoperative diagnosis and decision-making during mitral valve plasty.

The perioperative ASA/SCA guidelines show that the use of TEE during mitral valve plasty is indicated in category B2 cases, and that intraoperative assessment by TEE is essential to improving patient outcomes [2, 3]. On the other hand, various complications due to TEE manipulation have been reported. Namely, esophageal injury has been reported to occur in 0.9–1.2% of cases [4, 5]. Mitral valve plasty requires a more detailed evaluation of the valve by TEE and more frequent probe manipulation, which may injure tissues vulnerable to mechanical stimuli and increase complications. A previous study of TEE performed in postoperative patients with reflux esophagitis reported that TEE-related complications were rare (< 1%) [6]. However, there were other imaging limitations in this setting, such as the inability to insert an adult probe into the esophagus or to obtain transgastric and mid-esophageal images [6].

To prevent TEE-related complications, considerations include the ease and findings of upper gastrointestinal endoscopy prior to TEE, the use of pediatric probes, and minimizing probe manipulation [7]. Although conducting upper gastrointestinal endoscopy before TEE is controversial [8, 9], it may be useful for patients with preexisting esophageal and gastric lesions, such as the patient in this case. In postoperative patients with esophageal cancer, it is difficult to objectively evaluate the feasibility of TEE because the anatomy has been altered by resection and reconstruction of the oropharynx and stomach. For the purpose of evaluating the feasibility of TEE in postoperative patients with upper gastrointestinal cancer, preoperative upper gastrointestinal endoscopy should be considered. The lack of evidence for either cancer recurrence or stenosis of the gastrointestinal tract from the upper gastrointestinal endoscopy 2 years prior encouraged the use of intraoperative assessment by TEE in this case. Intraoperative complications of TEE could be avoided by gently manipulating the probe without forcing it into areas of resistance.

**Fig. 3:**
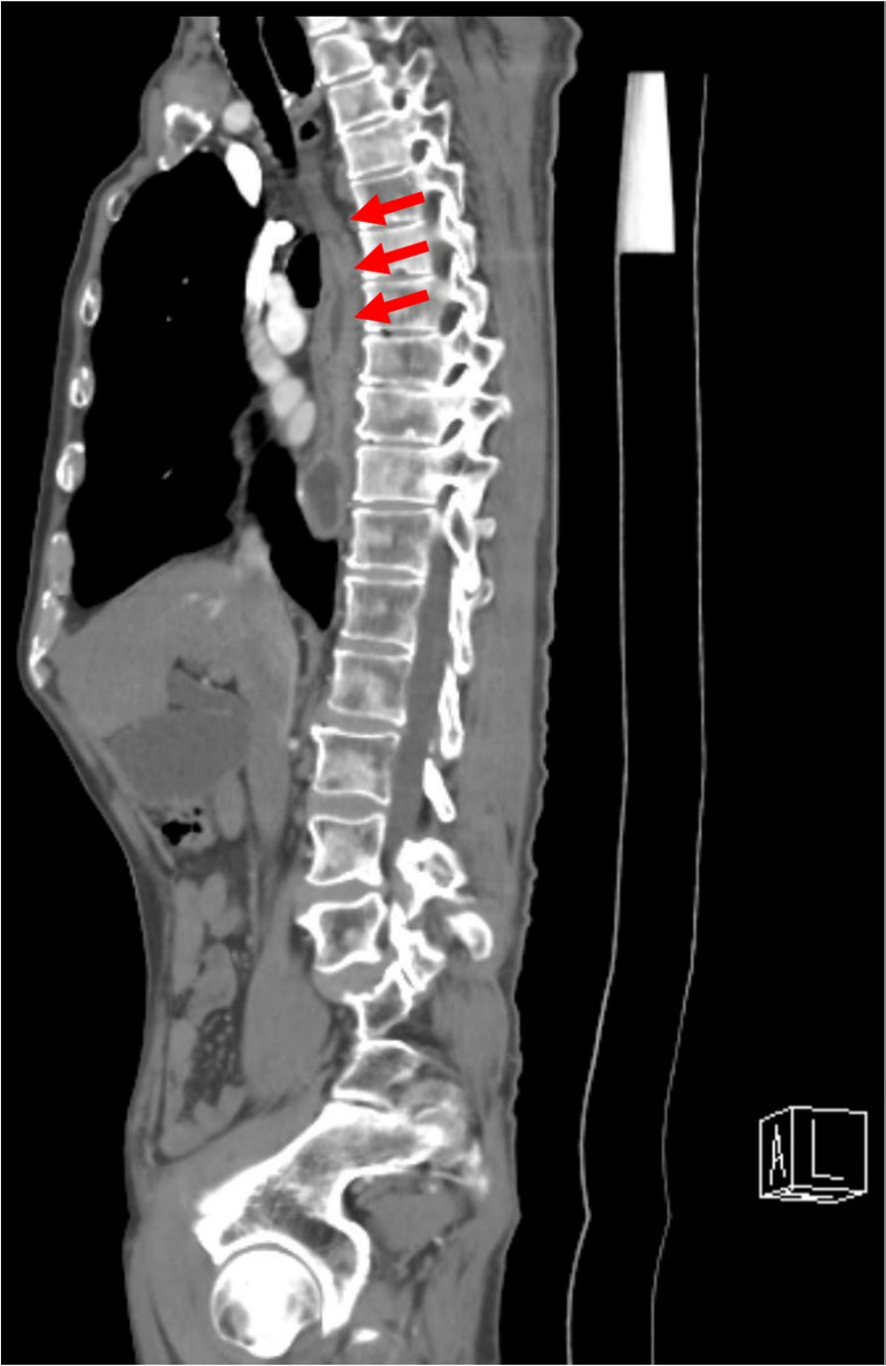
Preoperative computed tomography. The esophagus was reconstructed in the posterior mediastinal tract (red arrows), and there was no finding of recurrence

TEE was useful in determining the success of mitral valve repair despite the limitations of probe manipulation in the gastrointestinal tract. Gastrointestinal tract function after esophageal cancer surgery has been reported to be similar to preoperative gastric acid secretion and motility [10, 11]. In this case, the TEE probe could not proceed in the area where the esophagus was reconstructed (Fig. 3). This might be because the gastric tube was wider and more elastic than the normal esophagus, and the TEE probe did not advance and sticks to it, even if the preoperative upper gastrointestinal endoscopy did not show cancer recurrence or stenosis. The TEE image improved as surgery progressed, and this was attributed to the improved fitting between the probe and the reconstructed gastrointestinal tube with gastric secretions. TEE in this case was useful for mitral valve surgery in that it enabled a more detailed evaluation of the left ventricular system and left auricle than epicardial echography. However, while the mitral valve could be assessed, a normal transgastric or more distally oriented image could not be obtained due to the limited progression of the probe. Therefore, the possibility of complications during withdrawal from CPB, such as coronary air embolization, need to be assessed without the use of TEE.

## Conclusion

Intraoperative TEE was useful for intraoperative decision-making during mitral valve plasty in the remote stage after posterior mediastinal canal reconstruction for esophageal cancer. In cases where the benefits outweigh the risks, such as in mitral valve surgery, TEE should be considered with careful attention to the possibility of complications.

## Data Availability

Not applicable.
