# Correction: Experience with transesophageal echocardiography for mitral valve plasty in the remote stage after esophagectomy with gastric tube reconstruction via the posterior mediastinal route

**DOI:** 10.1186/s40981-023-00651-7

**Published:** 2023-09-04

**Authors:** Yuri Sato, Takaharu Tokita, Junichi Saito, Kazuyoshi Hirota

**Affiliations:** 1 Department of Anesthesiology, Aomori Prefectural Central Hospital, 2-1-1 Higashitsukurimichi, Aomori, 030-8553 Japan; 2 Department of Anesthesiology, Hirosaki University Graduate School of Medicine, 5 Zaifu-Cho, Hirosaki, 036-8562 Japan

**Correction: JA Clin Rep 9, 45 (2023)**

**https://doi.org/10.1186/s40981-023-00625-9**

Following publication of the original article [1], the author reported that there is an error in the article title. The word “retrosternal” should be “**posterior mediastinal**”

This has been corrected above and the original article has been updated.
